# Fifth generation telepathology systems. Workflow analysis of the robotic dynamic telepathology Component

**DOI:** 10.1186/1746-1596-8-S1-S3

**Published:** 2013-09-30

**Authors:** BL Braunhut, AR Graham, LC Richter, PD Webster, EA Krupinski, AK Bhattacharyya, RS Weinstein

**Affiliations:** 1 Departments of Pathology and Medical Imaging and Arizona Telemedicine Program, University of Arizona College of Medicine, Tucson, AZ, USA

## Background

Fifth generation telepathology systems are dual-modality systems (i.e., digital pathology systems) that combine whole slide imaging (WSI) and real-time dynamic robotic telepathology. Hybrid robotic dynamic/static image telepathology systems were the precursors of fifth generation telepathology systems and can be used as surrogates for fifth generation systems in workflow studies [1-4].

Variability in human performance was identified as a pathology issue in the first scientific paper on telepathology, published in 1987 [5]. In a study performed under highly controlled conditions, it was shown that individual pathologists had a range of thresholds for diagnosing breast cancer on frozen sections. The use of the “equivocal for malignancy” diagnostic category varied significantly among the six pathologists enrolled in an Receiver Operating Characteristic (ROC) video microscopy human performance study. Surprisingly, use of this category was essentially the same for both conventional light microscopy and video microscopy for each pathologist [5]. In another early study, Dunn *et al.*, using a hybrid dynamic robotic/static image telepathology system, documented patterns of telepathology primary diagnoses case deferrals for seven telepathologists over a 12-year period of time. These seven telepathologists rendered provisional primary surgical pathology diagnoses on over 11,000 surgical pathology cases. Case deferral rates among the telepathologists varied from 2.5% to 32.7% [6,7].

In this workflow study, we compared the QA case deferral rates of general pathologists and subspecialty surgical pathologists staffing a telepathology-based QA program. Fifth generation telepathology systems are an extension of the hybrid dynamic robotic/static image system concept, replacing the gallery of individual static images with a single large whole slide imaging (WSI) file.

## Materials and methods

### Telepathology-based quality assurance service

UMC initiated a QA telepathology consultation service between Lake Havasu City, Arizona and Tucson, Arizona (approximately 300 miles away) in July 2005. The Arizona Telemedicine Program (ATP) provided the broadband telecommunications infrastructure for the service as well as a telepathology case coordinator. These QA services continued uninterrupted until October 2009 when HRMC changed direction and decided to outsource all of its laboratory services to a commercial reference laboratory for cost savings.

During this study, the HRMC pathology laboratory handled 3,000-4,000 surgical pathology cases per year and was locally staffed by a single pathologist. Surgical cases at HRMC were accessioned, grossed, embedded in paraffin and glass slides were produced in the HRMC laboratory. All new cancer cases and any other challenging surgical pathology cases were identified by the HRMC pathologist for telepathology QA review.

QA cases reviewed via telepathology had been diagnosed with a written report generated by the HRMC pathologist prior to telepathology. A remotely controllable hybrid robotic-dynamic telepathology system (Apollo PACS ®, Falls Church, VA) was used to transmit a stream of digital images, via the ATP network, from Lake Havasu City, Arizona to Tucson, Arizona. The case video images were reviewed in real-time and remotely navigated by the on-service triage telepathologist at UMC, in Tucson. The telepathologist, linked to the HRMC pathologist in Lake Havasu City, was able to collaborate face-to-face via real-time videoconferencing, a feature built into the Apollo system. At the time of each telepathology review, the HRMC pathology report and accompanying patient medical history/demographic data were made available to the telepathologist in Tucson.

**Figure 1:**
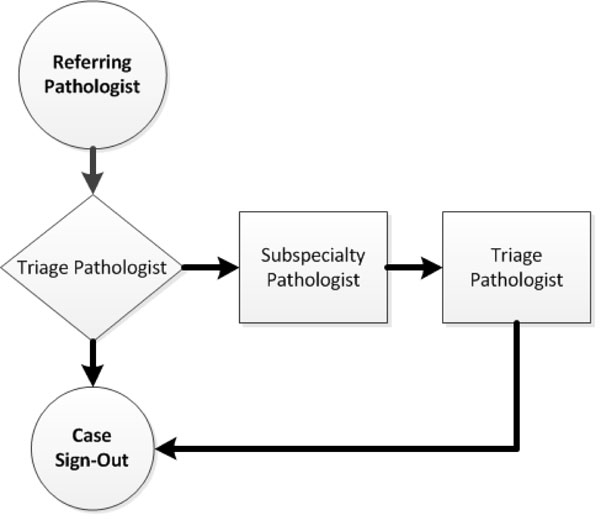
**CTP workflow model** Workflow for the Case Triage Practice (CTP) Model used in the Lake Havasu-Tucson, Arizona, telepathology quality assurance service. When triage pathologists consult with subspecialty pathologists, they retain responsibility for generating the final QA telepathology report. The service used an Apollo hybrid dynamic robotic/static image telepathology system. (Adapted from Bhattacharyya et al., 1995 [8]).

Ten UMC telepathologists participated in the study. They had dual roles, functioning either as general telepathologists and/or as subspecialty surgical pathologists depending on the nature of the case. Nine surgical pathology subspecialties were represented, including dermatopathology, gastrointestinal/hepatic, renal/genitourinary, breast, thoracic, gynecologic and head/neck pathology. The CTP case workflow model, as described by Bhattacharyya *et al.*, is shown schematically in Figure 1 [8].

### Study cases and data analysis

Between July 2005 and October 2009, 1862 cases were transmitted from HRMC to UMC.

After completion of case accruals, data analysis commenced. The provisional surgical pathology reports (HRMC) and the final telepathology case reports (UMC) were compiled. Data compiled included date of telepathology review, name of telepathologist, specimen organ system, preliminary diagnosis, and final telepathology diagnosis. These data were uploaded into a Microsoft Excel spreadsheet and tallied for case load per telepathologist, number of cases per organ system, and deferral rates. The HRMC and UMC diagnoses were compared and classified by a senior pathologist as being either concordant or discordant. Discordant diagnoses were further sub-classified by the staff pathologist as a major discrepancy (one that would alter clinical management) or a minor discrepancy (one that would not change clinical management).

Cases were further evaluated with regards to the telepathologist’s area of subspecialty surgical pathology expertise. A t-test for paired observations was used to determine if there was a statistically significant difference in deferral rates and a Chi-square test was done to determine if the distributions of deferral rates differed across readers (null hypotheses=deferral rate expected to be the same).

## Results and discussion

Of the 1815 analytical cases, 1650 cases (90.91%) were signed out directly by the triage telepathologist. The remaining 165 cases (9.09%) were deferred for further analysis by a subspecialty surgical pathologist or for special studies such as immunohistochemistry.

### Levels of concordance

Overall concordance was 94.27%. Concordance by specimen site/organ system ranged from 90.09% to 100%. The greatest discordance rate was seen with genitourinary cases with an overall discordance rate of 9.91% (7.66% major discrepancies, 2.25% minor discrepancies). Concordance on breast cases, cardiovascular cases, and head and neck cases was 100%. Table 1 includes 42 cases which were initially viewed by two telepathologists.

**Table 1:** Concordance of HRMC and UMC diagnoses. Table 1 includes 42 cases that were initially viewed by two telepathologists.

Organ/site	Cases	Agree	Major disc.	Minor disc.
Gastrointestinal	494	474 (95.95%)	8 (1.62%)	12 (2.43%)
Genitourinary	444	400 (90.09%)	34 (7.66%)	10 (2.25%)
Skin	246	230 (93.50%)	5 (2.03%)	11 (4.47%)
Lungs	240	235 (97.92%)	1 (0.42%)	4 (1.67%)
Bone/soft tissue	87	81 (93.10%)	1 (1.15%)	5 (5.75%)
Head/neck	63	63 (100%)	0 (0%)	0 (0%)
Gynecological	49	45 (91.84%)	0 (0%)	4 (8.16%)
Breast/axilla	38	38 (100%)	0 (0%)	0 (0%)
Endocrine	21	19 (90.48%)	0 (0%)	2 (9.52%)
Cardiovascular	10	10 (100%)	0 (0%)	0 (0%)
				
Total	1692	1595 (94.27%)	49 (2.90%)	48 (2.83%)

### Deferral rates

The case volumes per telepathologist ranged from 51 to 501 cases (average 182 cases). Deferral rates for individual telepathologists ranged from 4.79% to 21.26% (average 10.05%). Deferral rates were minimally changed by exclusion of cases within each telepathologist’s subspecialty area and ranged from 4.94% to 21.81% (average 10.26%). These data are summarized in Table 2. A t-test showed no statistically significant difference in deferral rates for case triage telepathologists for cases outside their areas of subspecialty pathology expertise versus triage cases falling within their area of subspecialty surgical pathology expertise (t = 0.032, p = 0.9754). However, 8 out of 10 telepathologists deferred a lower percentage of telepathology cases that fell within their area of surgical pathology expertise which may represent a trend (Table 2). A Chi-square test for distribution of deferral rates across readers was statistically significant for general rates (X^2^ = 20.52, p < 0.05) and subspecialty rates (X^2^ = 20.23, p < 0.05).

**Table 2:** Deferral rates

Pathologist	Total cases	Deferred cases	Total cases excluding pathologists subspecialty	Total deferred cases excluding pathologists subspecialty	Deferral rate overall	Deferral rate of cases within pathologists subspecialty	Deferral rate excluding pathologists subspecialty
Pathologist A	501	24	344	17	4.79%	4.46%	4.94%
Pathologist B	369	30	321	25	8.13%	10.42%	7.78%
Pathologist C	188	24	150	22	14.79%	5.26%	14.67%
Pathologist D	174	37	165	36	21.26%	11.11%	21.81%
Pathologist E	166	12	161	12	7.23%	0%	7.45%
Pathologist F	139	12	109	10	8.63%	6.67%	9.17%
Pathologist G	85	9	83	9	10.59%	0%	10.84%
Pathologist H	84	6	76	6	7.14%	0%	7.89%
Pathologist I	58	7	50	5	12.07%	25%	10%
Pathologist J	51	4	50	4	7.84%	0%	8%

This study evaluated a real-time telepathology QA program using the CTP case workflow model.[8] The overall concordance rate between primary (HRMC) and final (UMC) diagnosis was 94.27%. Of the discordant diagnoses, 2.90% represented major discrepancies and 2.83% represented minor discrepancies. In clinical practice, this discordance rate could be of concern [9,10].

Overall discordance rates were minimally changed with exclusion of cases within each telepathologist’s subspecialty area. Conversely, UMC subspecialty surgical pathologists performed well in the role of general triage pathologist (Figure 1). This supports the use of subspecialty surgical pathologists as the general triage pathologist in a telepathology-based QA program.

## Conclusion

This CTP workflow telepathology model, blending the services of university-based subspecialty surgical pathologists and a community-based general pathologist, provided a means for improving the quality of community-based laboratory services, presented opportunities for UMC-based community outreach, and increased the efficiency of a second opinion QA program. Service provider and user satisfaction were high.

Based on these data and observations, we suggest that the likelihood of a reviewing telepathologist agreeing or disagreeing with a diagnosis rendered at an outside hospital is not a function of the reviewers’ expertise alone, but rather may be related to other human factors, even possibly the personality type of the telepathologist. For individual pathologists, there was a strong relationship between rates of case deferrals for cases within their own area of subspecialty expertise as compared with cases they handled that were outside of their area of subspecialty expertise. Low, intermediate, and high level users of the “case deferral” option could be identified. It would be of interest to conduct Myers-Briggs personality assessments on pathologists and to correlate personality assessment results with other quantifiable pathologist performance measures such as surgical pathology case deferral rates, rates of equivocation on malignant diagnoses, and others.

## List of abbreviations

ATP: Arizona Telemedicine Program; CTP: Case Triage Practice; HRMC: Havasu Regional Medical Center; QA: quality assurance; SPP: subspecialty pathology practice; UMC: University Medical Center; WSI: whole slide imaging.

## Competing interests

Dr. Weinstein is Scientific Director and has stock options in Apollo PACS®.

## Authors’ contributions

BB compiled, analyzed and reviewed data sets, and authored the first draft of the manuscript. AG reviewed, edited, and composed portions of the manuscript. LR collected data and provided technical support for the original telepathology consultation sessions. PW was clinical coordinator for the study and assisted with data compilation. EK reviewed the data and performed the statistical analysis. AB was Laboratory Medical Director overseeing the telepathology consultation service and provided telepathology support. RW conceived the study, authored sections of the manuscript, and provided telepathology expertise. All authors read and approved the final manuscript.
